# Imaging in Vulval Cancer

**DOI:** 10.3390/cancers16122269

**Published:** 2024-06-19

**Authors:** Minah Ha, Lois Eva

**Affiliations:** Department of Gynaecological Oncology, Te Toka Tumai Auckland City Hospital, Auckland 1023, New Zealand; minahh@adhb.govt.nz

**Keywords:** vulval cancer, diagnostic imaging, ultrasound, CT, PET/CT, MRI

## Abstract

**Simple Summary:**

Vulval cancer is a rare gynaecological cancer, accounting for 3% of all gynaecological malignancies. The aim of this publication was to review the current evidence for the role of imaging in the diagnosis and staging of vulval cancer. We found that there is insufficient evidence to support the routine use of imaging for the assessment of primary vulval tumours. For nodal staging, there is no ideal imaging modality that shows superiority over other modalities. For the assessment of distant metastases, CT CAP and PET/CT have the most evidence supporting their use.

**Abstract:**

Vulval cancer is a rare gynaecological cancer, accounting for 3% of all gynaecological malignancies, with 47,000 cases in 2022 globally. Various imaging modalities are widely used in conjunction with clinical assessment in the diagnosis and staging of vulval cancers; however, there is significant heterogeneity in which modalities are recommended in international guidelines, reflecting the paucity of evidence in this area. We reviewed the current evidence for the role of imaging in vulval cancer. A systematic search of the literature was performed on the PubMed database using the MeSH terms ‘vulval neoplasm’ and ‘diagnostic imaging’. We found that there is insufficient evidence to support the routine use of imaging for primary vulval tumours. For nodal assessment, there is no ideal imaging modality with sensitivity or specificity that is superior to other modalities. For distant metastases, CT CAP and FDG-PET/CT have the most evidence to support their use. In conclusion, the evidence for role of imaging in vulval cancer is limited by the heterogeneity of the study design and diagnostic criteria used in each study and the small sample size and retrospective nature of most studies.

## 1. Introduction

Vulval malignancy is a rare gynaecological neoplasm accounting for 3% of all gynaecological malignancies, with 47,000 cases in 2022 globally [1]. Squamous cell carcinoma (SCC) is the most common histological diagnosis, constituting more than 90% of cases, followed by melanoma, adenocarcinoma, basal cell carcinoma, sarcoma and undifferentiated type [2]. Around 59% of vulval cancers present early with localised disease, while 30% and 6% of cases have metastatic disease in regional lymph nodes and distant sites, respectively [3]. The standard treatment is radical resection of the primary tumour and surgical staging of the inguino-femoral lymph nodes [4]. An important prognostic factor in vulval cancer is lymph node status; the disease-free survival rate at two years is 88% in node-negative patients and 60%, 43% and 29% in patients with one, two or more than two positive lymph nodes, respectively [5]. Accurate preoperative staging is therefore crucial in treatment planning for vulval cancers.

The aim of this review is to evaluate the current evidence in imaging aspects of vulval carcinoma, in its diagnosis, staging, treatment planning, surveillance and in the setting of suspected recurrence. A systematic search of the literature was performed on the PubMed database, using the MeSH terms ‘vulval neoplasm’ and ‘diagnostic imaging’. Sixty-two manuscripts written in the English language were reviewed.

The most widely used staging system for vulval malignancy is the International Federation of Gynaecology and Obstetrics (FIGO) staging system, most recently revised in 2021 based on analyses of prospectively collected data and validated prognostic capability [6]. This is shown in Table 1. Important notations and changes include specifying lymph node positivity as micrometastasis and macrometastasis and allowing the incorporation of cross-sectional imaging findings into vulval cancer staging, similar to cervical cancer [7]. FIGO staging applies to all morphological types of vulval malignancy, with the only exception being vulval melanoma.

## 2. Summary of Current Guidelines

There is considerable variation in recommendations for imaging in vulval cancer in different guidelines, reflecting the paucity of prospective data in this area.

European Society of Gynaecology Oncology (ESGO) guidelines recommend tailoring imaging modalities depending on the extent of the disease, including no imaging required for clinically FIGO stage IA disease, magnetic resonance imaging (MRI) for locally advanced tumours, ultrasound (US) of the groin for assessment of inguino-femoral lymph nodes, and whole-body computed tomography (CT) for metastasis [8]. For surveillance, it is recommended that imaging should only be performed based on risks of recurrence, symptoms or findings suggestive of recurrence. Groin US may be considered for surveillance in node-negative patients treated with sentinel lymph node (SLN) dissection; however, comment is made on the lack of proven benefit/cost-effectiveness. For suspected recurrence, CT, MRI, chest/abdomen/pelvis fluoro-D-glucose positron emission tomography/CT (FDG-PET/CT) or PET-MRI is recommended for assessing the vulval, groin and pubic area, as well as for detecting possible metastases. ESGO guidelines are only applicable to SCC of the vulva and do not address other vulval cancer histologies.

The National Comprehensive Cancer Network (NCCN) guidelines published in 2024 recommend consideration of chest imaging with chest X-ray and pelvic MRI for initial workup, with other imaging modalities guided by symptomatology and clinical concern for metastatic disease [4]. FDG-PET/CT may be considered in patients with positive sentinel lymph nodes, for evaluation of residual nodal disease in the groin or pelvis, which may guide additional treatment. For follow-up, FDG-PET/CT could be considered at 3–6 months to assess treatment response after definitive primary treatment, but otherwise, imaging should be based on symptoms and clinical concern for recurrent or metastatic disease. For suspected recurrence/metastasis, whole-body CT or FDG-PET/CT should be considered, with pelvis MRI to aid in further treatment planning if confirmed recurrence/metastasis. These recommendations apply to SCC and adenocarcinoma of the vulva, with separate recommendations for vulval melanoma.

The European Society of Urogenital Radiology (ESUR) guidelines published in 2021 recommend no routine imaging for stage IA disease, in line with ESGO guidelines [9]. Pelvic MRI is recommended for local staging of SCC with stromal invasion > 1 mm, tumour size > 4 cm, or tumours with suspicious involvement of the urethra, vagina or anus (i.e., FIGO stages IB and II). For tumours > 2 cm and ≤4 cm, clinical staging and groin US (with fine needle aspiration (FNA) cytology of suspicious lymph nodes) or MRI are recommended. For regional or locally advanced disease (FIGO stages III-IVA) or metastatic disease (FIGO stage IVB), chest/abdomen/pelvis CT (CT-CAP) or FDG-PET/CT is recommended, with intravenous contrast on portal-venous phase to increase diagnostic accuracy. For suspicion of recurrence, pelvis MRI is recommended for determining the extent of local recurrence, and restaging by CT-CAP or FDG-PET/CT is recommended for groin recurrence. Similar to ESGO guidelines, these recommendations apply to SCC of the vulva and not to other histological types of vulval SCC.

The Society of Gynaecologic Oncology (SGO) published a guideline specifically on post-treatment surveillance and diagnosis of recurrence in gynaecologic malignancies in 2017 [10]. For vulval malignancy, routine use of radiographic imaging for surveillance is not recommended due to a lack of evidence to support this. In the setting of suspected recurrence, CT-CAP or FDG-PET/CT is recommended, especially if exenterative surgery is considered, to rule out distant disease.

## 3. Imaging Modalities for the Primary Vulval Tumour

There are only a few studies that have examined the role of imaging for the primary lesion in vulval malignancies, reflecting the critical value of thorough clinical examination.

There are two retrospective studies evaluating the role of MRI in assessing the extent of the primary lesion. In a retrospective study published in 2002, the staging accuracy of pelvic MRI was evaluated in 22 patients with primary vulval SCC undergoing surgery, with an accuracy of 70% [11]. Similar results were seen in another retrospective study by Kataoka et al., where diagnostic accuracy of pelvic MRI was assessed in 36 patients with primary vulval cancer, with 69% accuracy for the primary tumours [12].

The use of FDG-PET/CT was evaluated in two retrospective studies. Peiro et al. showed that the sensitivity of FDG-PET/CT for the detection of vulval malignancy was 100% in SCC and 60% in non-squamous lesions in their retrospective study of 10 patients [13]. This was consistent with the 100% sensitivity of FDG-PET/CT for detecting primary tumours in a retrospective study of 47 patients by Yanarates et al. [14].

Wessels et al. evaluated the use of optical coherence tomography (OCT) for determining surgical margins for SCC of the vulva [15]. In OCT, backscattered light is used to produce cross-sectional images, similar to backreflected sound waves in ultrasonography. In their prospective study of 18 patients undergoing surgery for vulval SCC, OCT was shown to have a sensitivity of 100% and specificity of 80% when a threshold of 0.35 mm was used to distinguish between benign (<0.35 mm) and (pre)malignant lesions (>0.35 mm).

## 4. Imaging Modalities for Nodal Assessment

In contrast to the paucity of evidence for imaging for primary vulval lesions, there are several prospective and retrospective studies evaluating imaging modalities in assessing inguino-femoral lymph node status in vulval cancer. This reflects the poor sensitivity of clinical examination in detecting inguino-femoral lymph node metastases [16], and the high rate of postoperative morbidity associated with radical groin dissection, necessitating a non-invasive or minimally invasive technique that allows the selection of patients for whom unnecessary groin lymphadenectomy can be avoided [17].

### 4.1. Magnetic Resonance Imaging (MRI)

Six studies have reviewed the utility of MRI in assessing nodal status (Table 2) [11,12,18,19,20,21]. Within these six studies, there were significant heterogeneity in the diagnostic criteria for metastatic lymph nodes and considerable differences in the reported sensitivity and specificity of MRI in detecting metastatic lymph nodes (Table 2). Two studies reported a low sensitivity of 40–52% [11,20], while the other four studies reported a high sensitivity of 86–89% [12,18,19,21]. Specificity was consistently reported high, ranging from 82 to 100%, except for Sakae et al. reporting a specificity of 70.6%. All six studies had a small sample size, ranging between 10 and 60 patients.

### 4.2. Computed Tomography (CT)

Four studies evaluating the role of CT-CAP in the preoperative assessment of lymph node metastases were identified (Table 3) [22,23,24,25]. Most of these studies used a nodal diameter > 10 mm as a diagnostic criterion, with abnormal appearance and evidence of necrosis as other markers of lymph node metastases on CT. All four studies reported a low sensitivity of 43–58% and specificity of 75–96% and concluded that preoperative CT is of limited value for preoperative diagnosis of nodal metastases.

### 4.3. Positron Emission Tomography/Computed Tomography (PET/CT)

PET/CT is the modality most widely studied for its usefulness in assessing nodal status in vulval cancer (Table 4). The majority of studies, however, had a small sample size ranging from 8 to 25 patients, with only one study having a significantly large sample size of 160 patients. In an early study by De Hullu et al., PET CT using L-[1–^11^C]-tyrosine (TYR) as a tracer was evaluated in 25 patients with vulval cancer, with a low sensitivity of 53%. It was concluded that TYR-PET was not superior to palpation in detecting inguinal lymph node metastases [26]. All other studies examining the role of PET-CT used fludeoxyglucose-18 (^18^F-FDG) as the tracer, with conflicting results. A number of studies reported a high sensitivity of 92–100% and concluded that ^18^F-FDG PET/CT may be a useful modality in the preoperative assessment of lymph node status in vulval cancers [27,28,29,30]. Conversely, a number of other studies have reported a low sensitivity ranging from 50 to 80%, and concluded that ^18^F-FDG PET/CT is not accurate in predicting lymph node metastasis [31,32,33].

Most studies have consistently reported on high specificity and negative predictive value, with some authors concluding that PET may be useful in planning radiation treatment and as an adjunct to lymphoscintigraphy and sentinel lymph node biopsy [31,32,34]. In a retrospective study by Robertson et al., the physician’s prognostic impression changed in 54% of cases after PET/CT, suggesting that it may play an important role in treatment planning [35].

In 2021, Triumbari et al. published a systematic review and meta-analysis of 10 studies evaluating ^18^F-FDG PET/CT in vulval cancer patients [36]. In their analysis, qualitative per-patient analysis resulted in a pooled sensitivity of 70%, specificity of 90%, PPV of 86% and NPV of 77%. It was concluded that a negative preoperative PET/CT may exclude groin metastases and select patients with early vulval cancer, who may be eligible for a less radical surgical treatment.

**Table 4:** Summary of studies evaluating the role of PET/CT in the detection of inguino-femoral lymph node metastases.

Author	Type of Study	Year	Number of Patients	Diagnostic Criteria	Findings
De Hullu et al. [26]	Prospective	1999	25	Visual (qualitative) analysis	Sensitivity 53%
					Specificity 95%
					PPV 33%
					NPV 98%
Cohn et al. [31]	Prospective	2002	15	Visual (qualitative) analysis	Sensitivity 80%
					Specificity 90%
					PPV 80%
					NPV 90%
Kamran et al. [32]	Retrospective	2014	20	Focal increased tracer uptake (SUV_max_)	Sensitivity 50%
					Specificity 100%
					PPV 100%
					NPV 57%
Peiro et al. [13]	Retrospective	2014	10	Visual (qualitative) analysis	Sensitivity 100%
Lin et al. [27]	Prospective	2015	23	SUV_max_ interpreted by two nuclear medicine physicians and two radiologists	Sensitivity 92%
					specificity 91%
					PPV 85%
					NPV 95%
Dolanbay et al. [30]	Prospective	2015	8	Visual (qualitative) analysis	Sensitivity 100%
					Specificity 100%
Robertson et al. [35]	Retrospective	2016	54	Qualitative assessment	Change in prognosis in 51% of patients
Collarino et al. [29]	Prospective	2017	33	Qualitative (visual) analysis and semi-quantitative analysis (SUV_max_)	Sensitivity 95%
					Specificity 78%
					PPV 69%
					NPV 96%
Crivellaro et al. [33]	Retrospective	2017	29	Focal increased tracer uptake	Sensitivity 50%
					Specificity 67%
					PPV 58%
					NPV 59%
Garganese et al. [34]	Prospective	2017	47	Focal uptake of FDG tracer	Sensitivity 56%
					Specificity 88%
					PPV 38%
					NPV 93%
Oldan et al. [37]	Retrospective	2018	21	SUV_max_ cutoff of 4.5 or of two times the average liver uptake	Sensitivity 100%
					specificity 89%
Rufini et al. [28]	Retrospective	2021	160	Visual assessment	Sensitivity 86%
					Specificity 66%
					PPV 52%
					NPV 91%
				Semi-quantitative analysis (SUV_max_ cut-off 1.89)	Sensitivity 73%
					Specificity 85%
					PPV 68%
					NPV 88%
				Overall assessment	Sensitivity 79%
					Specificity 78%
					PPV 61%
					NPV 89%
Triumbari et al. [36]	Systematic review and meta-analysis	2021	72	As per individual studies	Sensitivity 70%
					Specificity 90%
					PPV 86%
					NPV 77%

### 4.4. Ultrasound (US)

Ultrasound (US) assessment of lymph node status was studied in several small studies (Table 5). Similar to studies evaluating MRI and CT, there was significant heterogeneity in how a positive lymph node was defined on US. Most studies reported a high sensitivity and specificity, in the ranges of 80–100% and 83–96%, respectively, with improved accuracy when combined with FNA cytology [22,38,39,40,41,42]. Some authors have concluded that US, combined with FNA cytology, may be a useful preoperative tool to identify those who need groin dissection from those with uninvolved nodes, who could be spared from groin dissection and the associated morbidity [39,40]. Conversely, De Gregorio et al. conclude that, due to the fatality of a missed nodal metastasis, surgical staging for nodal metastases is still required, despite the relatively high sensitivity and specificity of US in predicting lymph node metastases [41]. Findings from a study by Sykes et al. further support the importance of accurate surgical staging and pathological assessment of sentinel lymph nodes, and not relying on imaging alone to detect nodal metastases [43]. In their multicentre retrospective review, a critical evaluation was performed on cases with early-stage vulval cancer with negative sentinel lymph nodes that later developed groin recurrence. It was found that in two of these cases, the recommended pathology protocol had not been adhered to, with metastases identified following serial sectioning of the node. In view of this evidence, the role of imaging is to exclude nodal metastases, not to diagnose them.

In 2022, Verri et al. published a systematic review and meta-analysis of eight studies on US assessment of groin lymph nodes in vulval cancer, some of which have been described above [44]. The pooled sensitivity and specificity from the meta-analysis were 85% and 86%, respectively. The pooled PPV and NPV were 65% and 92%, respectively. The authors concluded that ultrasound should be considered an accurate tool for assessment of nodal staging, to allow patients with low risk of nodal metastases to receive less invasive surgeries such as sentinel node biopsy; however, further prospective multicentre studies are required to confirm these findings.

### 4.5. Sentinel Lymph Node Biopsy

Sentinel lymph node identification using lymphoscintigraphy and the use of technetium-99 m-labelled nanocolloid (99 mTc) has also been evaluated in a number of studies [45,46,47,48,49,50,51,52,53,54,55]. Eleven of these studies were reviewed in a systematic review published in 2005, with a pooled sensitivity of 97% and a specificity of 100% [56].

The safety and clinical utility of the sentinel lymph node procedure were studied in GROINSS V-I, which was a multicentre observational study of 403 patients [57]. Patients with negative sentinel lymph nodes had no further treatment, while patients with positive sentinel lymph nodes had full inguino-femoral lymphadenectomy, and postoperative external radiation therapy was performed in those with more than one intranodal metastasis and/or extranodal tumour growth. The groin recurrence rate was 2.3% in patients with unifocal vulval disease and negative sentinel lymph nodes, with a median follow-up of 35 months.

Intraoperative use of indocyanine green (ICG) coupled with near-infrared (NIR) fluorescence imaging as a potential alternative to radioisotope use has been assessed in a number of studies, due to the high cost and complex logistics associated with radioisotope use [58]. A systematic review of 13 studies assessing the use of ICG and NIR fluorescence for the detection of SLN reported a detection rate of 89.7–100%, with no study demonstrating the superiority of other detection techniques compared to ICG and NIR imaging [59].

## 5. Imaging Modalities for Distant Metastasis

There is limited literature on the best imaging modality when assessing the presence of distant metastases in vulval cancer. The two modalities that have been studied are CT CAP and FDG-PET/CT.

Andersen et al. reviewed the role of preoperative CT CAP in 27 patients with localised vulval cancer [23]. No distant metastases were found, and preoperative CT did not change the initial treatment plan for any of the patients. Robertson et al. reviewed the addition of PET/CT to the conventional CT or MRI imaging in 54 patients and found that distant metastases were found in 10 patients, 4 of which were not seen on CT-CAP [35]. In a retrospective review by Lin et al., FDG PET/CT was reviewed in 23 patients with vulval cancer, in addition to CT-CAP or MRI [27]. There were no significant differences in sensitivity, specificity or accuracy between CT or MRI and FDG-PET/CT. Peiro et al. reviewed the utility of FDG-PET/CT in 10 patients, with four studies with possible distant metastases; 3 of these patients had changes to their management based on their FDG-PET/CT findings [13].

## 6. Imaging Modalities for Surveillance after Treatment and for Suspected Recurrent Vulval Cancer

Only two studies specifically evaluating imaging for surveillance and for suspected recurrence were identified in our review [60,61].

Pouwer et al. reviewed the role of groin US in women with negative sentinel lymph nodes in their single-centre prospective study [60]. In total, 139 women with vulval SCC with a negative SLN were monitored with three monthly ultrasounds of the groin. Two asymptomatic isolated groin recurrences were detected, after 348 ultrasounds and 29 FNA biopsies in total, with a sensitivity of 100% and specificity of 92%. The authors concluded that routine follow-up with ultrasound of the groin may lead to early detection of asymptomatic isolated groin recurrence.

In a retrospective multicentre study by Albano et al., the impact of FDG-PET/CT on treatment decision making in 63 patients with suspected recurrent vulval cancer was reviewed [61]. Out of 63 patients, 52 had the presence of recurrence on PET/CT, with reported sensitivity, specificity, PPV, NPV and accuracy of 100%, 92%, 98%, 100% and 98%. In 28 patients, PET/CT was registered as having a relevant impact; 12 moved from local therapy to systemic treatment due to disseminated disease, 10 showed the site of recurrence that was negative on conventional imaging, and 6 cases were confirmed to be true negatives and avoided unnecessary treatment. Based on these results, the authors have concluded that PET/CT has a high sensitivity and specificity in assessing patients with recurrent vulval cancer, with a significant impact on clinical decision making.

## 7. Conclusions

The role of imaging in vulval cancer has evolved over time. As with all rare tumours, the evidence for the role of imaging in vulval cancer is limited by the heterogeneity of the study design and diagnostic criteria used in each study, as well as the small sample size and retrospective nature of most studies. In early-stage disease (i.e., FIGO stage IA), an accurate assessment with clinical examination of the primary tumour may suffice for staging prior to treatment. In disease where the depth of invasion of the tumour is more than 1 mm, imaging has an important role in the assessment of local spread and in detecting nodal and distant metastases. In terms of delineation of local disease, MRI has superior soft-tissue resolution over other modalities and gives the best assessment of the size of the primary tumour, as well as the involvement of surrounding structures. For nodal staging, there is no perfect imaging modality that shows superiority over other modalities. Given the non-inferiority of US compared with CT, MRI or FDG-PET/CT, as well as the wider availability and low cost of US, further prospective studies on optimising the diagnostic performance of US may yield the most value in advancing our preoperative assessment of nodal status. Regardless of the modality used, consistent and comprehensive reporting on nodal assessment, such as that suggested by the Vulvar International Tumour Analysis (VITA) group, is crucial for optimising our diagnostic capability of nodal staging [62]. CT-CAP is the most useful in the detection of distant metastases and recurrent disease, especially if combined with FDG-PET/CT.

## Figures and Tables

**Table 1:** FIGO 2021 staging for carcinoma of the vulva [6].

Stage	Description	
I	Tumour confined to the vulva	
	IA	Tumour size ≤ 2 cm and stromal invasion ≤ 1 mm ^a^
	IB	Tumour size > 2 cm or stromal invasion > 1 mm ^a^
II	Tumour of any size with extension to lower one-third of the urethra, lower one-third of the vagina, lower one-third of the anus with negative nodes	
III	Tumour of any size with extension to upper part of adjacent perineal structures, or with any number of non-fixed, non-ulcerated lymph node	
	IIIA	Tumour of any size with disease extension to upper two-thirds of the urethra, upper two-thirds of the vagina, bladder mucosa, rectal mucosa, or regional lymph node metastases ≤ 5 mm
	IIIB	Regional ^b^ lymph node metastases > 5 mm
	IIIC	Regional ^b^ lymph node metastases with extracapsular spread
IV	Tumour of any size fixed to the bone, or fixed, ulcerated lymph node metastases, or distant metastases	
	IVA	Disease fixed to pelvic bone, or fixed or ulcerated regional lymph node metastases
	IVB	Distant metastases

^a^ Depth of invasion is measured from the basement membrane of the deepest, adjacent, dysplastic, tumour-free rete ridge (or nearest dysplastic rete peg) to the deepest point of invasion. ^b^ Regional refers to inguinal and femoral lymph nodes.

**Table 2:** Summary of studies evaluating the role of MRI in the detection of inguino-femoral lymph node metastases.

Author	Type of Study	Year	Number of Patients	Diagnostic Criteria	Findings
Sohaib et al. [11]	Retrospective	2002	22	>10 mm short axis for superficial LN	Sensitivity 40%
					Specificity 97%
				>8 mm short axis for deep LN	Sensitivity 50%
					Specificity 100%
Hawnaur et al. [18]	Prospective	2002	10	One of the following: long axis diameter > 21 mm, short axis diameter > 10 mm, long/short axis ratio < 1.3:1, irregularity of contour, cystic changes within solid parts of an LN	Sensitivity 89%
					Specificity 91%
Singh et al. [19]	Retrospective	2006	59	Two of the following: short axis diameter 1 cm, irregular or rounded shape, increased signal intensity on STIR or heterogenous signal intensity on T2-WI	Sensitivity 86%
					Specificity 82%
Bipat et al. [20]	Prospective	2006	60	Overall impression based on short axis diameter ≥8 mm, size in axial/sagittal and coronal plane, localisation, appearance, margin and shape	Sensitivity 52%
					Specificity 85%
					PPV 46%
					NPV 87%
Kataoka et al. [12]	Retrospective	2010	49	Overall impression based on long and short axis diameter, contour, presence of cystic changes/necrosis, loss of fatty hilum, signal intensity similar to primary lesion	Sensitivity 88%
					Specificity 86%
					PPV 88%
					NPV 86%
Sakae et al. [21]	Retrospective	2016	41	Long axis > 10 mm	Sensitivity 88%
					Specificity 71%
					PPV 58%
					NPV 92%

PPV: positive predictive value. NPV: negative predictive value.

**Table 3:** Summary of studies evaluating the role of CT-CAP in the detection of inguino-femoral lymph node metastases.

Author	Type of Study	Year	Number of Patients	Diagnostic Criteria	Findings
Land et al. [22]	Retrospective	2006	44	Long axis ≥ 10 mm, evidence of necrosis in an LN, or evidence of extranodal disease	Sensitivity 58%
					Specificity 75%
					PPV 58%
					NPV 75%
Andersen et al. [23]	Prospective	2015	27	Short axis > 10 mm and/or abnormal contrast enhancement	Sensitivity 60%
					Specificity 90%
					PPV 38%
					NPV 96%
Pounds et al. [24]	Prospective	2020	116	Not specified	Sensitivity 59%
					Specificity 78%
					PPV 62%
					NPV 76%
Bohlin et al. [25]	Retrospective	2021	134	Short axis > 10 mm, abnormal shape, attenuation or contrast enhancement	Sensitivity 43%
					Specificity 96%
					PPV 88%
					NPV 73%

**Table 5:** Summary of studies evaluating the role of US and FNA cytology in the detection of inguino-femoral lymph node metastases.

Author	Type of Study	Year	Number of Patients	Diagnostic Criteria	Results
Makela et al. [16]	Prospective	1993	25	Nodal hypoechogenicity, absence of internal hilar echoes, greatest diameter > 1.5 cm, roundish shape, thickness ratio length > 1/2	Sensitivity 82%
					Specificity 87%
Moskovic et al. [38]	Prospective	1999	24	LN with a more circular or irregular configuration and with loss of central hilar fat	Sensitivity 85%
					Specificity 83%
				FNA cytology	Sensitivity 58%
					Specificity 100%
				US combined with FNA cytology	Sensitivity 83%
					Specificity 82%
Mohammed et al. [39]	Prospective	2000	20	Short axis > 8 mm	Sensitivity 83%
					Specificity 90%
					PPV 63%,
					NPV 97%
				Long/short axis ratio ≤ 2	Sensitivity 100%
					Specificity 58%
					PPV 38%
					NPV 100%
				Short axis > 8 mm AND L/S axis ratio ≤ 2	Sensitivity 83%
					Specificity 88%
					PPV 63%
					NPV 96%
Hall et al. [40]	Prospective	2003	44	Grading based on size, shape (L/S ratio), preservation of an echogenic hilum, general attenuation and vascularity on Doppler	Sensitivity 86%
					Specificity 96%
				FNA cytology	Sensitivity 75%
					Specificity 100%
				Combined US + FNA cytology	Sensitivity 93%
					Specificity 100%
Land et al. [22]	Retrospective	2006	44	Circular or irregular configuration, loss of central hilar fat	Sensitivity 87%
					Specificity 69%
					PPV 48%
					NPV 94%
				US features plus FNA cytology on the largest or most abnormal node	Sensitivity 80%
					Specificity 100%
					PPV 100%
					NPV 93%
De Gregorio et al. [41]	Retrospective	2013	60	Absence of fatty hilum, irregular shape, cortical region diameter ≥ 4 mm, peripheral vascularisation	Sensitivity 76%
					Specificity 91%
					PPV 83%
					NPV 88%
Garganese et al. [42]	Retrospective	2020	144	Cortical thickness > 2.5 mm	Sensitivity 90%
					Specificity 78%
					PPV 59%
					NPV 96%
				Short axis > 8.5 mm	Sensitivity 64%
					Specificity 91%
					PPV 74%
					NPV 86%
				Cortex/medulla (C/M) ratio > 1.2	Sensitivity 70%
					Specificity 92%
					PPV 73%
					NPV 90%
				Combination of short axis and C/M ratio	Sensitivity 89%
					Specificity 82%
					PPV 68%
					NPV 95%
				Final overall assessment	Sensitivity 86%
					Specificity 84%
					PPV 73%
					NPV 92%
Verri et al. [44]	Systematic review and meta-analysis	2022	437	As per individual studies	Sensitivity 85%
					Specificity 86%
					PPV 65%
					NPV 92%

## Data Availability

No new data were created or analysed in this study. Data sharing is not applicable to this article.
